# Several dementia subtypes and mild cognitive impairment share brain reduction of neurotransmitter precursor amino acids, impaired energy metabolism, and lipid hyperoxidation

**DOI:** 10.3389/fnagi.2023.1237469

**Published:** 2023-08-16

**Authors:** Roberto Aquilani, Matteo Cotta Ramusino, Roberto Maestri, Paolo Iadarola, Mirella Boselli, Giulia Perini, Federica Boschi, Maurizia Dossena, Anna Bellini, Daniela Buonocore, Enrico Doria, Alfredo Costa, Manuela Verri

**Affiliations:** ^1^Department of Biology and Biotechnology, “Lazzaro Spallanzani,” University of Pavia, Pavia, Italy; ^2^Unit of Behavioral Neurology and Center for Cognitive Disorders and Dementia, IRCCS C. Mondino Foundation, Pavia, Italy; ^3^Dementia Research Center, IRCCS Mondino Foundation, Pavia, Italy; ^4^Department of Biomedical Engineering of the Montescano Institute, Istituti Clinici Scientifici Maugeri IRCCS, Montescano, Italy; ^5^Neurorehabilitation Unit of the Montescano Institute, Istituti Clinici Scientifici Maugeri IRCCS, Montescano, Italy; ^6^Department of Drug Sciences, University of Pavia, Pavia, Italy; ^7^Department of Brain and Behavioral Sciences, University of Pavia, Pavia, Italy

**Keywords:** dementias, mild cognitive impairment, cerebrospinal fluid amino acid precursors of neurotransmitters, energetic substrates, oxidative stress

## Abstract

**Objective:**

Dementias and mild cognitive impairment (MCI) are associated with variously combined changes in the neurotransmitter system and signaling, from neurotransmitter synthesis to synaptic binding. The study tested the hypothesis that different dementia subtypes and MCI may share similar reductions of brain availability in amino acid precursors (AAPs) of neurotransmitter synthesis and concomitant similar impairment in energy production and increase of oxidative stress, i.e., two important metabolic alterations that impact neurotransmission.

**Materials and methods:**

Sixty-five demented patients (Alzheimer’s disease, AD, *n* = 44; frontotemporal disease, FTD, *n* = 13; vascular disease, VaD, *n* = 8), 10 subjects with MCI and 15 control subjects (CTRL) were recruited for this study. Cerebrospinal fluid (CSF) and plasma levels of AAPs, energy substrates (lactate, pyruvate), and an oxidative stress marker (malondialdehyde, MDA) were measured in all participants.

**Results:**

Demented patients and subjects with MCI were similar for age, anthropometric parameters, biohumoral variables, insulin resistance (HOMA index model), and CSF neuropathology markers. Compared to age-matched CTRL, both demented patients and MCI subjects showed low CSF AAP tyrosine (precursor of dopamine and catecholamines), tryptophan (precursor of serotonin), methionine (precursor of acetylcholine) limited to AD and FTD, and phenylalanine (an essential amino acid largely used for protein synthesis) (*p* = 0.03 to <0.0001). No significant differences were found among dementia subtypes or between each dementia subtype and MCI subjects. In addition, demented patients and MCI subjects, compared to CTRL, had similar increases in CSF and plasma levels of pyruvate (CSF: *p* = 0.023 to <0.0001; plasma: *p* < 0.002 to <0.0001) and MDA (CSF: *p* < 0.035 to 0.002; plasma: *p* < 0.0001). Only in AD patients was the CSF level of lactate higher than in CTRL (*p* = 0.003). Lactate/pyruvate ratios were lower in all experimental groups than in CTRL.

**Conclusion:**

AD, FTD, and VaD dementia patients and MCI subjects may share similar deficits in AAPs, partly in energy substrates, and similar increases in oxidative stress. These metabolic alterations may be due to AAP overconsumption following high brain protein turnover (leading to phenylalanine reductions), altered mitochondrial structure and function, and an excess of free radical production. All these metabolic alterations may have a negative impact on synaptic plasticity and activity.

## 1. Introduction

Dementia is a syndrome that is characterized by reduced cognitive function and development of neuropsychiatric symptoms (NPS) (Cummings et al., 2022), including agitation, aggression, irritability, elation, disinhibition, altered motor behavior, appetite changes, depression, sleep disturbances, and symptoms of psychosis (Cerejeira et al., 2012). Up to 90% of demented patients experience one or more NPS over the course of the disease (Cummings et al., 2022).

Alterations in cerebral neurotransmitter systems, which contribute to the development of NPS manifestations and dementia progression (Vermeiren et al., 2013), are often present in combination in all dementias and in subjects with mild cognitive impairment (MCI). Indeed, combined alterations in brain neurotransmitter systems, including adrenergic- (dopa, dopamine, norepinephrine), serotonergic-, and cholinergic signaling occur in Alzheimer’s disease dementia (AD), frontotemporal dementia (FTD), vascular dementia (VaD), and MCI (Cummings et al., 2022).

In addition to deficits in cholinergic signaling (the main altered neurotransmitter system) (Grothe et al., 2013; Scheltens et al., 2016; Lammers et al., 2018), AD patients exhibit dysfunctional dopamine transmission in the hippocampus (Xiang et al., 2017), and impaired noradrenergic and dopaminergic pathways in the dorsolateral and anterior prefrontal cortex (Vermeiren et al., 2014). In the hippocampus of AD depressed patients, the serotonergic system is impaired, whereas in the same area of AD aggressive patients, deficient noradrenergic and serotonergic signaling pathways are observed (Vermeiren et al., 2014).

Studies on the abnormal functioning of the adrenergic system in FTD patients are inconsistent, having reported reduced cerebrospinal fluid (CSF) dopamine levels (Sjögren et al., 1998), increased dopa levels, not only in CSF but also in Brodmann’s area 46 (Janssens et al., 2020), and higher dopamine levels in the prefrontal cortex at post-mortem (Vermeiren et al., 2016). These findings may indicate the heterogeneity of the FTD population in terms of perturbed adrenergic neurotransmitter pathways. In FTD, while the noradrenergic system is normal or even increased in the frontal lobe (Vermeiren et al., 2016), serotonin transmission is reduced and is associated with several behavioral symptoms (Huey et al., 2006). Levels of serotonin catabolism are the same as in control subjects (Janssens et al., 2020).

Although FTD patients have reduced cholinergic neurons in the nucleus basalis, the cholinergic pathways to the cortex are preserved, indicating that the cholinergic damage in FTD is less pronounced than in AD.

Vascular dementia is due to brain vascular damage as reported in investigations dealing with neuropathological (Toledo et al., 2013; Arvanitakis et al., 2016) and neuroimaging (Montagne et al., 2015; Van de Haar et al., 2016a,b; Kisler et al., 2017; Sweeney et al., 2018) CSF biomarkers (Iturria-Medina et al., 2016; Sweeney et al., 2018). Cerebral vessel pathology contributes to approximately 50% of all dementias, including AD (Wardlaw et al., 2013; Blair et al., 2015; Iadecola, 2017; Montagne et al., 2017; Bennett et al., 2018; Sweeney et al., 2018).

Deficiencies in the serotonergic system have been reported in both acute stroke patients with depression symptoms (Rocco et al., 2007) and in later phases of stroke recovery (Pinto et al., 2017), and have been confirmed in patients in the hyperacute phase (<4.5 h) (Saccaro et al., 2022) of central infarction and in patients after 1 to 90 days from index event. As subacute (<3 months from the onset of symptoms) stroke patients may exhibit low plasma tyrosine (Tyr), the amino acid precursor of brain adrenergic compounds (Aquilani et al., 2004), VaD patients might have disturbances in the adrenergic pathway.

Abnormalities in neurotransmitter pathways occur in subjects with MCI (Peter et al., 2021), the transitional phase between aging and dementia (Petersen, 2011; Morris, 2012). Cholinergic system alterations occurring in atrophied basal forebrain (BF) nuclei (Mesulam et al., 1983; Mesulam, 2004; Mufson et al., 2007; Grothe et al., 2010, 2013; Muth et al., 2010; Peter et al., 2021) lead to altered modulation of both cortical activity and cognitive functions, more specifically memory and attention (Hasselmo and Sarter, 2011).

Considering that all the considered dementia subtypes and MCI almost always involve reductions of cholinergic, adrenergic, and serotonergic signaling, this may represent a minimum common denominator in all the diseases.

The rationale of the current study was that the reduced formation of brain neurotransmitters could be ascribed to a lack of availability of their amino acid precursors (AAPs), including methionine (Met), a precursor of choline (an essential substrate for acetylcholine, ACh synthesis), tyrosine (Tyr), necessary for dopa/dopamine/norepinephrine formation, and tryptophan (Trp), used for serotonin synthesis. In addition, we hypothesized that AD, FTD, and VaD patients, and MCI subjects could share similar reductions of CSF AAPs. Both hypotheses were based on the following reasons.

First, the above dementia subtypes and MCI may have increased brain AA overutilization from increased protein metabolism (Aquilani et al., 2022) and deposition of altered protein aggregates (Lee et al., 2001; Mariani et al., 2006; Hu and Grossman, 2009; Pendlebury and Rothwell, 2009; Bell et al., 2010). This leads to a reduction in the concentration of the essential amino acid phenylalanine (Phenyl-), which is greatly used for protein synthesis (Liu et al., 2014). Second, demented patients and MCI subjects may share one or more manifestations of NPS, particularly depression (Gabryelewicz et al., 2007), caused and/or favored by deficits in dopaminergic and serotonergic signaling.

Third, induction of a specific behavioral alteration involves the combination of several neurotransmitters (Vermeiren et al., 2013).

The last hypothesis we put forward was that AD, FTD, VaD, and MCI may share perturbed brain energy metabolism (Ferreira et al., 2010; Verri et al., 2012) and increased oxidative stress, as indirectly indicated by altered CSF levels of lactate, pyruvate and malondialdehyde (MDA), respectively. Given the crucial role played by adequate synapse energy availability and protein synthesis for synaptic transmission and neurotransmitter release, this is the main reason for determining energy substrates and oxidative stress (Todorova and Blokland, 2017). Moreover, it is well-known that an excess of free radicals negatively impacts cell and synapsis functions (Brawek et al., 2010).

Therefore, in this prospective observational study, CSF and plasma AAPs, Phenyl-, lactate, pyruvate, and MDA were measured in AD, FTD and VaD patients, and in MCI subjects and compared with a group of non-demented non-cognitively impaired subjects.

## 2. Materials and methods

### 2.1. Population

This prospective observational study included patient populations from our “Dementias database” National Institute of Neurology IRCCS Mondino Foundation, Pavia Italy. The selection criteria were Alzheimer’s disease (AD) diagnosis, frontotemporal neurodegeneration (FTD) and cerebrovascular disease (VaD). Moreover, subjects with a diagnosis of mild cognitive impairment (MCI) were selected. Among these populations, only demented patients and MCI subjects with available plasma and cerebrospinal fluid (CSF) levels of amino acids (AAs), lactate and pyruvate, and malondialdehyde (MDA) were considered.

Summarized below are the main characteristics of the demented patients and MCI subjects and the procedures they underwent.

The various dementia subtypes were cognitively impaired at the Mini Mental State Examination (MMSE scores): AD 16.24 ± 6.43; FTD 22.25 ± 6.44; VaD 20.73 ± 6.34. In MCI subjects, MMSE scores were 24.90 ± 3.00.

Both demented patients and MCI subjects underwent diagnostic testing in the Department of General Neurology at the National Institute of Neurology IRCCS Mondino Foundation, Pavia, Italy between 2014 and 2018.

Patients with dementia had a clinical dementia rating (CDR) ≥1 (Morris, 1993) and received an etiological diagnosis of typical AD (McKhann et al., 2011), behavioral variant of frontotemporal dementia (bvFTD) (Rascovsky et al., 2011), or vascular dementia (VaD) (Román et al., 1993). All patients with non-vascular dementia had a score <4 on the Modified Hachinski Ischemic Scale (Hachinski et al., 1975). The diagnosis of MCI was made according to the following criteria: (1) cognitive concern reflecting a change in cognition reported by patient, informant or clinician (i.e., historical or observed evidence of decline over time); (2) objective evidence of cognitive decline with cognitive test scores that are typically 1 to 1.5 standard deviations below the mean for their age and education matched peers on culturally appropriate normative data, CDR 0.5; and (3) preservation of independence in functional abilities (Albert et al., 2011).

Control subjects (CTRL) were selected from patients who were hospitalized in the same Department for reasons that were unrelated to cognitive disorders or inflammatory diseases. They were 11 males and 4 females, aged 73.6 ± 6.3 years with normal body weight (body mass index, BMI-kg/m^2^ −25.4 ± 1.7).

All procedures complied with the ethical standards of human experimentation and with the Helsinki Declaration of 1975, as revised in 2008. The study was approved by the Local Ethical Committee of Ospedale San Raffaele-Istituto di Ricovero e Cura a Carattere Scientifico-Milano, Italy at the time of the previous study (Aquilani et al., 2020) (Project identification code: MAIR2016; ethical approval: 130/INT/2016; date: 8 September 2016).

Participants or their legal representatives provided written informed consent to participate in the study. No participant received financial compensation.

### 2.2. Procedures

#### 2.2.1. Clinical evaluation

All enrolled patients underwent complete clinical, neurological, and neuropsychological assessment and brain magnetic resonance imaging (MRI).

In addition to physical examination, routine blood tests, anthropometric measurements (body weight, BW in kg; height in cm; body mass index, BMI, in kg/m^2^; mid-arm circumference in cm), clinical evaluation, and assessment of patients’ nutritional state by means of Mini Nutritional Assessment (MNA) score (Vellas et al., 1999) were carried out. MNA score <17 denotes a state of malnutrition, a score between 17 and 24 denotes risk of malnutrition, and a score >24 identifies a normal nutritional state.

#### 2.2.2. β–amyloid, tau and p-tau measurements in CSF compartment

Lumbar puncture was performed at the level of the L3/L4 or L4/L5 intervertebral space, according to the standard procedure used in our clinic for patients with cognitive disorders. CSF samples were centrifuged for 10 min at 1800 × *g* at 4°C within 3 h of collection. The samples were then divided into aliquots of 0.5 mL in polypropylene tubes and stored at -80°C. Measurement of CSF Aβ42, t-tau, and p-tau181 was performed using chemiluminescence enzyme immunoassay (Lumipulse G600II, Fujirebio).

#### 2.2.3. Plasma and CSF AA measurements

In demented patients, MCI subjects, and CTRL, at 8 a.m. after 12 h of overnight fasting, blood samples were drawn from an antecubital vein and immediately delivered to the laboratory, where plasma was obtained from heparinized blood using centrifugation (800 × *g*, 15 min) to measure plasma AA levels. At the same time, 2 mL of CFS samples were drawn from the lumbar tract and immediately delivered to the laboratory. Both CSF and plasma AA levels were used to calculate the CSF/plasma (CSF/P) AA ratios.

#### 2.2.4. Assessment of AA concentrations

The concentration of AAs, both in CSF and plasma, was measured using an AminoQuant II amino acid analyzer, based on the HP 1090 HPLC system, with fully automated precolumn derivatization. Both ortho-phthalaldehyde (OPA) and 9-fluorenyl-methyl-chloroformate (FMOC) reaction chemistries were used for the derivatization of primary and secondary AAs, respectively. A total of 1 μL of sample was injected on the column and AAs separated by applying the gradient indicated by the manufacturer’s protocol. Absorbance was measured simultaneously at 338 and 262 nm. CSF and plasma AA concentrations were expressed as μmol/L. The measurements of the CSF and plasma AAs were carried out in triplicate by the same laboratory and the mean of the three measurements was adopted. The characteristics of the method were based both on precision and standardization properties (unpublished data): precision, relative standard deviation (RSD) was 1.13%; reliability (bias) was 10.37%; the lower limit of quantitation (LOQ) was 0.0055 μmol/L; the limit of detection (LOD) was 0.0016 μmol/L. For the measurements in triplicate, the intra-day variability (RSD) was 3.21% and the intervariability was 4.77%.

The concentration of the AAPs of adrenergic neurotransmitters (AAP tyrosine), serotonin (AAP tryptophan), ACh (AAP methionine), and phenylalanine were measured. For these AAPs and Phenyl-, the CSF/P ratios were calculated.

For the reasons mentioned above, other AAs that are important for neurotransmission such as glutamate, glycine, glutamine, and others will not be discussed in the current study.

#### 2.2.5. Plasma and CSF pyruvate, lactate, and malondialdehyde measurements

As a rule, all measurements were carried out in triplicate, and the mean of the three measurements was calculated and adopted.

The following metabolic substrates and stress markers were assessed both in plasma and CSF using a microplate spectrophotometer (BioTek ELx800).

*Pyruvate*. Pyruvate levels, expressed in μmol/mL, were measured using the “Pyruvate Colorimetric/Fluorometric Assay Kit” (BioVision Incorporated, Milpitas, CA, USA) according to the manufacturer’s instructions. High CSF pyruvate levels is a marker of neuronal impaired ATP production (Parnetti et al., 1995).

*Lactate*. Lactate levels, expressed in μmol/mL, were measured using the “Lactate Colorimetric Assay Kit II” (BioVision Incorporated, Milpitas, CA, USA) according to the manufacturer’s instructions.

The lactate/pyruvate ratio was calculated in both experimental groups and CTRL.

*Malondialdehyde.* Malondialdehyde (MDA), a naturally occurring product of lipid peroxidation, was determined as an oxidative stress marker. MDA concentrations, expressed in μmol/L, were measured using the “Cayman’s TBARS Assay Kit” (Cayman Chemical Company, Ann Arbor, MI, USA) according to the manufacturer’s instructions.

#### 2.2.6. Insulin and HOMA-IR

Among the blood variables, plasma insulin levels (μU/mL) were measured using the Cord-CT Radioimmunoassay Kit CIS (France) and Coat A Count Insulin (D.P.C., Los Angeles, CA, USA) commercial kits. Insulin resistance was calculated using the Homeostasis Model Assessment (HOMA; normal value <2.4) (Matthews et al., 1985; Solerte et al., 2004).

### 2.3. Objectives

The first objective of the study was to document possible CSF reductions in AAPs between demented patients, MCI subjects, and CTRL.

The second objective of the study was to document possible reductions in CSF phenylalanine levels and to understand whether the reductions were similarly shared between demented patients and MCI subjects.

### 2.4. Statistical analysis

Descriptive statistics are reported as mean ± SD for continuous variables, and as numbers (N) and percentages (%) for discrete variables. Between-group comparisons for continuous variables were carried out by one-way analysis of variance (ANOVA) and by the Chi-square test for categorical variables. A significant result from ANOVA was followed up by *post hoc* analysis for pairwise comparisons (Dunn-Sidak method). The association between couples of variables was assessed by the Spearman’s correlation coefficient. All tests were two-tailed. A *p*-value < 0.05 was considered statistically significant. All statistical analyses were carried out using the SAS/STAT statistical package, release 9.4 (SAS Institute Inc., Cary, NC, USA).

## 3. Results

### 3.1. General characteristics of the study populations

Table 1 shows that experimental groups (demented and MCI) and control subjects were similar in terms of age and anthropometric variables. Biohumoral parameters and biomarker concentrations in CSF were similar across the dementia subtypes and MCI, except for p-tau, which was higher in MCI than in FTD. Both demented groups and MCI subjects had a state of insulin resistance.

**TABLE 1:** General characteristics of the study populations.

Variable	NV	AD (*N* = 44)	FTD (*N* = 13)	VaD (*N* = 8)	MCI (*N* = 10)	CTRL (*N* = 15)	*p*
**Demographic variables**							
Age (years)	–	72.3 ± 7.6	69.9 ± 8.9	75.5 ± 9.3	71.0 ± 7.0	73.6 ± 6.3	0.51
Male gender (%)	–	52.3	53.8	62.5	50.0	73.3	0.68
Disease duration (months)	–	40.3 ± 37.6	22.2 ± 15.4	37.1 ± 25.7	22.8 ± 15.9	–	0.19
Education (years)	–	6.62 ± 3.60	10.30 ± 4.92	7.25 ± 1.50	8.67 ± 4.04	−*	0.08
**Anthropometric variables**							
Body weight (kg)	–	64.8 ± 13.0	62.1 ± 11.6	70.5 ± 14.2	63.1 ± 12.2	70.4 ± 7.1	0.29
Height (cm)	–	160.9 ± 10.3	158.3 ± 9.5	163.3 ± 10.1	161.7 ± 11.3	165.9 ± 4.3	0.29
Body mass index	22–25 kg/m^2^	25.1 ± 4.9	25.0 ± 4.2	27.2 ± 6.4	24.0 ± 3.0	25.4 ± 1.7	0.68
Mid-arm circumference	M 27.9 cm; F 15.7 cm	26.5 ± 3.2	26.3 ± 3.0	26.7 ± 4.1	26.1 ± 2.1	−*	0.97
**Biohumoral variables**							
Glucose	70–115 mg/dL	93.5 ± 27.9	84.3 ± 8.4	114.0 ± 46.5	92.4 ± 12.6	−*	0.11
Insulin	4–24 μU/mL	13.38 ± 5.71	10.37 ± 4.63	13.70 ± 3.78	10.72 ± 4.20	−*	0.18
HOMA-IR	<2.4	4.15 ± 2.18	3.01 ± 1.68	4.58 ± 1.94	2.86 ± 1.29	−*	0.08
Glycosylated hemoglobin	4.8–5.9%	5.73 ± 0.91	5.45 ± 0.62	6.10 ± 1.52	5.65 ± 0.61	−*	0.50
Total cholesterol	<200 mg/dL	186.6 ± 36.4	183.8 ± 34.9	168.0 ± 22.1	187.4 ± 33.9	−*	0.57
HDL cholesterol	*M* > 55 mg/dL; *F* > 65 mg/dL	55.7 ± 15.4	54.7 ± 19.7	54.9 ± 19.7	51.7 ± 13.5	−*	0.92
LDL cholesterol	<100 mg/dL	111.1 ± 28.9	107.9 ± 20.3	89.0 ± 27.0	109.0 ± 28.5	−*	0.24
Triglycerides	0–200 mg/dL	95.4 ± 34.6	122.1 ± 106.9	118.4 ± 64.5	123.7 ± 38.4	−*	0.29
Transferrin	200–360 mg/dL	225.5 ± 38.6	234.7 ± 29.2	222.0 ± 32.9	223.3 ± 24.3	−*	0.85
Iron	59–158 μg/dL	86.6 ± 25.0	86.1 ± 23.9	78.6 ± 27.5	91.3 ± 27.5	−*	0.77
Vitamin B12	191–663 pg/mL	303.5 ± 105.2	399.8 ± 221.4	283.9 ± 65.7	252.3 ± 105.4	−*	0.047^§^
Folate	3.1–17.5 ng/mL	6.77 ± 3.79	7.30 ± 3.45	6.11 ± 1.92	5.68 ± 1.25	−*	0.67
Creatinine	M 0.73–1.18 mg/dL; F 0.55–1.02 mg/dL	0.86 ± 0.23	0.90 ± 0.23	1.02 ± 0.28	0.94 ± 0.16	−*	0.29
Total proteins	6.6–8.7 g/dL	6.28 ± 0.51	6.25 ± 0.32	6.19 ± 0.35	6.43 ± 0.36	−*	0.68
Albumin	55–69%	56.9 ± 4.7	57.4 ± 3.9	58.8 ± 2.0	59.5 ± 4.7	−*	0.41
White blood cell count	4.00–10.00 × 10^3^/μL	6.51 ± 1.90	6.12 ± 0.87	6.38 ± 1.62	6.60 ± 1.86	−*	0.90
Red blood cell count	M 4.30–5.70 × 10^6^/μL; F 3.80–5.20 × 10^6^/μL	4.26 ± 0.42	4.27 ± 0.32	4.40 ± 0.39	4.29 ± 0.58	−*	0.88
Hemoglobin	M 13.2–17.3 g/dL; F 11.7–15.5 g/dL	13.03 ± 1.19	12.96 ± 1.12	13.46 ± 1.44	13.01 ± 1.55	−*	0.81
Erythrosedimentation rate	<15 mm/1sth	20.1 ± 21.0	14.7 ± 8.7	17.5 ± 12.3	15.2 ± 15.6	−*	0.76
**Biomarker concentrations in CSF**							
tau	<404 pg/mL	491.5 ± 405.9	308.9 ± 148.9	436.3 ± 503.5	657.0 ± 373.8	–	0.40
p-tau	<56.5 pg/mL	80.1 ± 34.0	50.5 ± 20.5	84.7 ± 46.3	105.7 ± 54.7	–	0.050
β-amyloid	>599 pg/mL	546.2 ± 341.5	689.5 ± 281.3	509.3 ± 204.7	676.2 ± 198.5	–	0.57
β-amyloid/tau	>1.6	1.88 ± 2.76	2.92 ± 1.99	4.10 ± 4.66	1.58 ± 1.41	–	0.42
β-amyloid/p-tau	>11.5	8.17 ± 7.49	15.91 ± 10.41	8.10 ± 5.87	9.93 ± 9.49	–	0.14

^§^ : vitamin B12 FTD vs. MCI *p* = 0.056 (borderline significant). *: missing values due to technical fault. AD, Alzheimer’s disease; FTD, frontotemporal dementia; VaD, vascular dementia; MCI, mild cognitive impairment; CTRL, control subjects; NV, normal values; HOMA-IR, HOmeostasis Model Assessment-Insulin Resistance; CSF, cerebrospinal fluid; M, male; F, female.

### 3.2. Plasma energy metabolic substrates (pyruvate, lactate), oxidative stress marker (MDA), AAPs Tyr, Trp, Met, and Phenyl-

The results showed that experimental groups, compared to CTRL, had altered levels of metabolic substrates, increased oxidative stress and similar AA levels. Indeed, in Table 2 lactate levels were similar between demented groups, MCI, and CTRL. In contrast, pyruvate in each dementia subtype and MCI was higher than in CTRL. Consequently, lactate/pyruvate ratios were lower in experimental groups than in CTRL.

**TABLE 2:** Plasma energy metabolic substrates (lactate, pyruvate) oxidative stress marker malondialdehyde (MDA), amino acid precursors (AAPs) tyrosine (Tyr), tryptophan (Trp), methionine (Met), and phenylalanine (Phenyl-) of the study populations.

Variable	AD (*N* = 44)	FTD (*N* = 13)	VaD (*N* = 8)	MCI (*N* = 10)	CTRL (*N* = 15)	*p*	*Post hoc*
**Energy metabolic substrates**							
Lactate (NV 0.4–2.0 μmol/mL)	1.65 ± 0.61	1.60 ± 0.54	1.87 ± 0.69	1.74 ± 0.50	1.38 ± 0.30	0.28	
Pyruvate (NV 0.042–0.130 μmol/mL)	0.15 ± 0.06	0.17 ± 0.05	0.19 ± 0.07	0.14 ± 0.05	0.06 ± 0.03	<0.0001	A
Lactate/Pyruvate ratio	12.20 ± 5.93	9.81 ± 3.11	10.93 ± 5.01	13.65 ± 5.31	29.07 ± 16.48	<0.0001	B
**Oxidative stress marker**							
Malondialdehyde (μmol/L)	10.80 ± 3.52	10.47 ± 2.83	13.38 ± 4.32	11.73 ± 2.70	3.62 ± 0.86	<0.0001	C
**Amino acid precursors**							
Tyrosine (μmol/L)	53.5 ± 10.5	55.3 ± 14.4	58.0 ± 16.0	52.4 ± 15.3	53.4 ± 12.8	0.89	
Tryptophan (μmol/L)	43.7 ± 8.5	44.9 ± 9.1	47.0 ± 12.0	47.8 ± 11.8	40.9 ± 6.5	0.49	
Methionine (μmol/L)	–	–	–	–	–	–	
Phenylalanine (μmol/L)	51.6 ± 9.0	55.4 ± 11.5	60.6 ± 11.3	53.4 ± 5.9	57.7 ± 9.7	0.10	

*Post hoc* comparisons (Dunn-Sidak). A: AD vs. CTRL *p* < 0.0001; FTD vs. CTRL *p* < 0.0001; VaD vs. CTRL *p* < 0.0001; MCI vs. CTRL *p* = 0.002. B: AD vs. CTRL *p* < 0.0001; FTD vs. CTRL *p* < 0.0001; VaD vs. CTRL *p* < 0.0001; MCI vs. CTRL *p* = 0.0002. C: AD vs. CTRL *p* < 0.0001; FTD vs. CTRL *p* < 0.0001; VaD vs. CTRL *p* < 0.0001; MCI vs. CTRL *p* < 0.0001. AD, Alzheimer’s disease; FTD, frontotemporal dementia; VaD, vascular dementia; MCI, mild cognitive impairment; CTRL, control subjects; NV, normal values.

Tyr and Trp were similar in each dementia subtype, MCI, and CTRL. For technical reasons, Met concentrations were not available. There was no difference in Phenyl- levels between experimental populations and CTRL.

Significantly higher levels of the oxidative stress marker (MDA) were found in demented patients and MCI subjects, compared to CTRL.

### 3.3. CSF energy metabolic substrates (pyruvate, lactate) oxidative stress marker (MDA), AAPs Tyr, Trp, Met, and Phenyl-

In the CSF compartment of demented patients and MCI subjects, compared to CTRL, altered energy substrate levels, increased marker of oxidative stress and lower AA levels were found. Indeed, in Table 3 demented patients (with the exception of AD), MCI subjects, and CTRL had similar levels of lactate in the CSF compartment. In AD, lactate was higher than in CTRL (*p* = 0.003). Pyruvate levels were elevated in each dementia subtype and in MCI, in comparison to CTRL. Consequently, lactate/pyruvate ratios were significantly reduced in demented groups and MCI, in comparison to CTRL.

**TABLE 3:** Cerebrospinal fluid (CSF) energy metabolic substrates (lactate, pyruvate) oxidative stress marker malondialdehyde (MDA), amino acid precursors (AAPs) tyrosine (Tyr), tryptophan (Trp), methionine (Met), and phenylalanine (Phenyl-) of the study populations.

Variable	AD (*N* = 44)	FTD (*N* = 13)	VaD (*N* = 8)	MCI (*N* = 10)	CTRL (*N* = 15)	*p*	*Post hoc*
**Energy metabolic substrates**							
Lactate (NV 1.0–2.2 μmol/mL)	1.45 ± 0.38	1.40 ± 0.50	1.40 ± 0.49	1.22 ± 0.23	1.01 ± 0.10	0.005	A
Pyruvate (NV 0.02–0.11 μmol/mL)	0.11 ± 0.05	0.12 ± 0.05	0.15 ± 0.07	0.09 ± 0.02	0.03 ± 0.01	<0.0001	B
Lactate/Pyruvate ratio	14.60 ± 6.85	12.81 ± 5.49	11.24 ± 5.09	13.55 ± 3.53	34.48 ± 13.91	<0.0001	C
**Oxidative stress marker**							
Malondialdehyde (μmol/L)	2.01 ± 0.84	2.12 ± 0.92	2.80 ± 1.59	2.57 ± 1.55	0.99 ± 0.40	0.00045	D
**Amino acid precursors**							
Tyrosine (μmol/L)	9.26 ± 3.51	7.39 ± 1.51	5.22 ± 2.47	6.48 ± 2.49	13.02 ± 7.10	0.00015	E
Tryptophan (μmol/L)	1.99 ± 1.31	2.27 ± 0.68	2.10 ± 0.87	2.56 ± 1.23	4.51 ± 1.88	<0.0001	F
Methionine (μmol/L)	2.54 ± 1.83	2.20 ± 1.01	3.55 ± 2.20	5.02 ± 0.97	4.55 ± 1.86	<0.0001	G
Phenylalanine (μmol/L)	10.92 ± 4.23	10.36 ± 3.03	9.92 ± 3.73	9.41 ± 2.01	17.04 ± 4.16	<0.0001	H

*Post hoc* comparisons (Dunn-Sidak). A: AD vs. CTRL *p* = 0.003. B: AD vs. CTRL *p* < 0.0001; FTD vs. CTRL *p* < 0.0001; VaD vs. CTRL *p* < 0.0001; MCI vs. CTRL *p* = 0.023. C: AD vs. CTRL *p* < 0.0001; FTD vs. CTRL *p* < 0.0001; VaD vs. CTRL *p* < 0.0001; MCI vs. CTRL *p* < 0.0001. D: AD vs. CTRL *p* = 0.011; FTD vs. CTRL *p* = 0.035; VaD vs. CTRL *p* = 0.003; MCI vs. CTRL *p* = 0.002. E: AD vs. CTRL *p* = 0.03; FTD vs. CTRL *p* = 0.004; VaD vs. CTRL *p* = 0.001; MCI vs. CTRL *p* = 0.002. F: AD vs. CTRL *p* < 0.0001; FTD vs. CTRL *p* = 0.0003; VaD vs. CTRL *p* = 0.003; MCI vs. CTRL *p* = 0.006. G: AD vs. CTRL *p* = 0.002; FTD vs. CTRL *p* = 0.005. H: AD vs. CTRL *p* < 0.0001; FTD vs. CTRL *p* = 0.0002; VaD vs. CTRL *p* = 0.003; MCI vs. CTRL *p* < 0.0001. CSF, cerebrospinal fluid; AD, Alzheimer’s disease; FTD, frontotemporal dementia; VaD, vascular dementia; MCI, mild cognitive impairment; CTRL, control subjects; NV, normal values.

The oxidative stress marker MDA was elevated in each dementia subtype and in MCI, in comparison to CTRL. The levels of MDA were not significantly different among the dementia subtypes and between these subtypes and MCI.

Tyrosine levels in AD (*p* = 0.03), FTD (*p* = 0.004), VaD (*p* = 0.001), and MCI (*p* = 0.002) were lower than in CTRL.

Tryptophan was also lower in demented patients and MCI subjects (from *p* = 0.006 to *p* < 0.0001), in comparison to CTRL.

No differences in Tyr and Trp were found in AD, FTD, VaD, and MCI groups.

Compared to CTRL, Met was lower in AD (*p* = 0.002) and FTD (*p* = 0.005) but not in VaD or in MCI.

Phenyl- concentrations in the experimental populations were significantly lower than in CTRL and were not different among demented subtypes and between demented subtypes and MCI.

### 3.4. CSF/plasma ratios (CSF/P)

The results showed lower CSF/P ratios of AAPs in experimental groups than in CTRL. Indeed, in Table 4, compared to CTRL, CSF/P Tyr, and CSF/P Trp were lower in both demented patients and MCI subjects. CSF/P Phenyl- was lower in demented patients and in MCI subjects (from *p* = 0.009 to *p* = 0.002).

**TABLE 4:** Cerebrospinal fluid (CSF)/plasma ratios (CSF/P) of AAPs and phenylalanine.

Variable	AD (*N* = 44)	FTD (*N* = 13)	VaD (*N* = 8)	MCI (*N* = 10)	CTRL (*N* = 15)	*p*	*Post hoc*
**AAPs**							
CSF/P Tyrosine	0.17 ± 0.06	0.14 ± 0.04	0.09 ± 0.05	0.12 ± 0.04	0.25 ± 0.08	<0.0001	A
CSF/P Tryptophan	0.05 ± 0.03	0.05 ± 0.02	0.04 ± 0.02	0.06 ± 0.04	0.10 ± 0.03	0.0005	B
CSF/P Methionine	–	–	–	–	–	–	
CSF/P Phenylalanine	0.22 ± 0.1	0.19 ± 0.1	0.16 ± 0.07	0.18 ± 0.05	0.32 ± 0.08	0.0007	C

*Post hoc* comparisons (Dunn-Sidak). A: AD vs. CTRL *p* = 0.012; FTD vs. CTRL *p* = 0.0008; VaD vs. CTRL *p* < 0.0001; MCI vs. CTRL *p* = 0.0002. B: AD vs. CTRL *p* = 0.0002; FTD vs. CTRL *p* = 0.004; VaD vs. CTRL *p* = 0.006; MCI vs. CTRL *p* = 0.039. C: AD vs. CTRL *p* = 0.009; FTD vs. CTRL *p* = 0.004; VaD vs. CTRL *p* = 0.002; MCI vs. CTRL *p* = 0.004. CSF, cerebrospinal fluid; AD, Alzheimer’s disease; FTD, frontotemporal dementia; VaD, vascular dementia; MCI, mild cognitive impairment; CTRL, control subjects; AAPs, Amino Acid Precursors of neurotransmitters: tyrosine for dopamine and catecholamines; tryptophan for serotonin; methionine for acetylcholine.

### 3.5. Correlations between plasma and CSF lactate, pyruvate, and MDA

The study found several correlations between plasma, CSF energy substrates and MDA. Indeed, plasma and CSF lactate levels were significantly associated in AD (*r* = 0.65, *p* < 0.0001) and in FTD (*r* = 0.57, *p* = 0.047), but not in CTRL, VaD or MCI. The levels of circulating pyruvate showed a direct correlation with CSF pyruvate in AD (*r* = 0.56, *p* = 0.0002) but not in CTRL, FTD, VaD, or MCI.

In summary (Table 5), in CSF, the three dementia subtypes and MCI shared similar reductions in Phenyl- and AAPs, except for Met, which was only lower in AD and FTD. Furthermore, dementia subtypes and MCI shared increased MDA and pyruvate. In contrast, increased lactate was only observed in AD. In the plasma compartment, dementia subtypes and MCI shared similar reductions in Phenyl- and similar increases in pyruvate and MDA concentrations. The lactate levels were the same in patients as they were in CTRL.

**TABLE 5:** Cerebrospinal fluid (CSF) neurotransmitter amino acid precursors (AAPs), phenylalanine, energy metabolic substrates (lactate and pyruvate), oxidative stress marker malondialdehyde (MDA) in the study populations.

Variables	AD	FTD	VaD	MCI
**AAPs**				
Tyrosine	↓	↓	↓	↓
Tryptophan	↓	↓	↓	↓
Methionine	↓	↓	=	=
Phenylalanine	↓	↓	↓	↓
**Energy substrates**				
Lactate	↑	=	=	=
Pyruvate	↑	↑	↑	↑
Lactate/Pyruvate ratio	↓	↓	↓	↓
MDA	↑	↑	↑	↑

↑Indicates increase in comparison to controls. ↓Indicates reduction in comparison to controls. =Indicates no significant changes in comparison to controls. CSF, cerebrospinal fluid; AD, Alzheimer’s disease; FTD, frontotemporal dementia; VaD, vascular dementia; MCI, mild cognitive impairment.

## 4. Discussion

The present study shows that patients with AD, FTD, and VaD, and subjects with MCI may share similar reductions in CSF AAPs of adrenergic, serotonergic, and cholinergic neurotransmitters. The shared reduction in AAP of the cholinergic neurotransmitter is limited to AD and FTD patients. Similarly, all groups shared increased brain protein metabolism (low CSF Phenyl-), perturbed cell aerobic metabolism (high CSF pyruvate), increased glycolytic activity (high CSF lactate, only found in AD), and cerebral cell lipid hyper-oxidation (high MDA). It is of interest to note that extra-cerebral protein metabolism was not different between experimental groups and CTRL, as suggested by similar plasma Phenyl- levels.

The study confirms the AAP defects that were previously observed in AD patients (Aquilani et al., 2022) and extends them to FTD, VaD, and MCI.

Low brain AAPs may negatively impact brain neurotransmission by impairing neurotransmitter formation (Murley and Rowe, 2018). The fact that MCI subjects and dementia patients shared similar Tyr and Trp deficits indicates that the reduced AAPs within brain structures may occur at an early stage of the progression from aging to MCI to dementia (Jongsiriyanyong and Limpawattana, 2018).

The absence of differences in Tyr or Trp precursors between MCI subjects and dementia patients is not surprising considering that several neurological and metabolic conditions show MCI to be more similar to AD than to the declined cognitive function that occurs in healthy aging. Indeed, MCI presents atrophy of medial temporal lobe areas and the posterior cingulate cortex (Fennema-Notestine et al., 2009), hypoperfusion of parietal cortices and the hippocampus (Habert et al., 2011), and hypometabolism in temporoparietal and posterior cingulate cortices (Kim et al., 2010). Moreover, lower CFS amyloid β (Aβ42), from poor brain clearance, and elevated CSF concentrations of total and phosphorylated tau, which have previously been reported in MCI (Anderson, 2019) are also confirmed in the current study. Interestingly, MCI patients showed higher p-tau synthesis than FTD, suggesting a higher brain anabolic activity.

### 4.1. Some potential mechanisms underlying reduced CSF AAPs

Several factors shared by AD, FTD, VaD, and MCI may explain the overutilization of amino acids in the brain, leading to lower AAPs in the CSF compartment. These factors include insulin resistance, the biosynthesis of neuropathological markers and the mutual influence of these two elements on each other (Aquilani et al., 2022).

Insulin resistance (Apelt et al., 1999; Bigl et al., 2003; Mazzola and Sirover, 2003; Freude et al., 2009; Ahmed et al., 2014, 2016; Cooper et al., 2015; Hong et al., 2021; Aquilani et al., 2022) reduces glucose utilization in mitochondria by blocking the mitochondrial pyruvate dehydrogenase enzyme complex (Lazar, 2002) thus leading to impaired energy generation. Given the brain’s inability to process fatty acids for energy production (Griffin and Bradshaw, 2017), an excess of AAs is used in the tricarboxylic acid cycle (TCA) as an energy substrate (Burns et al., 2010; Griffin and Bradshaw, 2017). The alterations of anaerobic glucose breakdown in AD patients were likely more pronounced than in the other dementia subtypes and in MCI, thus accounting for high CSF lactate. Translocated intestinal bacteria, bacterial amyloid, and toxins may increase brain inflammation, favoring the development of insulin resistance (Bhattacharjee and Lukiw, 2013; Friedland, 2015; Zhao et al., 2015; Aquilani et al., 2022).

Another mechanism increasing AAP utilization is promoted by the Aβ that can activate mTOR signaling (Li et al., 2005; Caccamo et al., 2011), which stimulates the synthesis of tau protein, the main component of the neurofibrillary tangles (Jahrling and Laberge, 2015), and mitochondrial activity (Schieke et al., 2006).

The interrelationship between insulin resistance and neuropathological markers may further explain AAP overutilization. Hyperinsulinemia increases the phosphorylation of tau proteins (Clodfelder-Miller et al., 2006; Planel et al., 2007), the formation of senile plaques (De la Monte and Wands, 2005), and inhibits the degradation of extraneuronal Aβ (Qiu et al., 1998). In turn, both Aβ and tau further impair brain insulin signaling (Biundo et al., 2018) (vicious cycle). Brain amino acid overutilization in FTD may follow increased synthesis of protein aggregates (Raz et al., 2016), including cellular p-tau (Lee et al., 2001) and FTD-transactive response DNA-binding protein-43 (TDP-43) (Davidson et al., 2007; Hu and Grossman, 2009).

In VaD brain tissue, AA overutilization may be due to increased amyloid synthesis and deposition (Bell et al., 2010). Amyloid formation is due to prolonged vasculotoxic and neurotoxic effects of vascular damage-induced hypoxia and blood-brain barrier permeability (Wardlaw et al., 2013; Iadecola, 2017; Sweeney et al., 2018; Nation et al., 2019). On the other hand, cerebral vessel disease contributes to approximately half of all dementias (Iadecola, 2017). In turn, Aβ42 deposition (Iadecola, 2017; Kisler et al., 2017; Montagne et al., 2017) and tau protein (Bennett et al., 2018) cause vascular damage. Vessel damage-induced ischemia leads to reduced oxygen supply and further reduction of monoamine neurotransmitters. Indeed, the syntheses of biogenic amines are very sensitive to a reduced oxygen supply, as tyrosine dehydrolase and tryptophan hydroxylase activities need molecular oxygen for catalyzing the syntheses of monoamine neurotransmitters (Frazer and Hensler, 1999; Kuhar et al., 1999). Previous studies have documented that vascular damage contributes to cognitive decline (Zlokovic, 2011; Snyder et al., 2015; Gottesman et al., 2017; Iadecola, 2017; Sweeney et al., 2018).

It is of clinical interest to note that individuals with early cognitive impairment may have brain capillary damage and BBB disruption in the hippocampus, independently of neuropathologic marker deposition (Nation et al., 2019).

Brain amino acid overutilization from increased protein synthesis may also occur in subjects with MCI following the upregulation of brain genes associated with anabolic processes and mitochondrial energy generation in synaptic genes (Berchtold et al., 2014).

In the current study, the reduction of CSF Met, a precursor of choline, which is the essential substrate for ACh synthesis, was only found in AD and FTD cohorts. This suggests that VaD patients and MCI subjects have a better conservation of the cholinergic system, although MCI subjects had a reduced forebrain, the pacemaker of cholinergic transmission (Peter et al., 2021). We speculate that the choline formed from Met could ensure the formation of ACh, thus limiting the negative impact of basal forebrain atrophy. Maintaining ACh generation as far as possible is important because ACh released under the activity of cholinesterase supplies choline to neurons (Taylor and Brown, 1999).

It is important to consider that another source of brain choline is the phosphatidylcholine catabolism (Taylor and Brown, 1999).

### 4.2. Energy metabolic substrates and oxidative stress

In the current study, the high levels of pyruvate both in plasma and CSF, together with the finding of normal plasma lactate concentrations, low lactate/pyruvate ratios in FTD, VaD, and MCI suggest that mitochondrial dysfunction is more prevalent than dysfunction in the glycolytic pathway in the brain and extracerebral districts. The current investigation confirms a previous study reporting high CSF levels of pyruvate in AD and VaD (Parnetti et al., 2000). The derangements of both cell mitochondrial structure and enzymes leading to impaired energy production (ATP) have been reviewed in several studies (Mosconi et al., 2008; Ferreira et al., 2010; Verri et al., 2012). The high CSF and plasma pyruvate levels and low lactate/pyruvate ratios in experimental groups indicate that (1) the deficit of energy production (hypometabolism) (Liguori et al., 2015, 2016) is due to combined defects of anaerobic and aerobic metabolism energy-producing in brain and extra-brain organs/tissues; and (2) the alterations of the aerobic pathway of energy generation are more pronounced than the alterations of the anaerobic metabolism.

The high lactate in AD patients is in line with previous investigations reporting elevated CSF lactate (Liguori et al., 2015, 2016).

The high MDA levels found in all the experimental groups analyzed in the current study suggest that abnormal mitochondrial activity is responsible for the increased generation of free radicals and the development of a state of oxidative stress leading to cell membrane lipolysis. The lipid hyper-oxidation can have a devastating impact on brain and extra brain cell integrity due to an excess of free radicals that results in cell lysis (Sharp et al., 2004; Huerta-Alardín et al., 2005), contributing to brain and extra brain tissue atrophy.

High plasma and CSF pyruvate indicates an inefficient aerobic pathway of energy generation. Reduced pyruvate utilization in TCA is likely to be an effect of cell insulin resistance. Another important negative effect of low pyruvate utilization in the TCA cycle is the reduction of choline formation and consequently ACh synthesis (Taylor and Brown, 1999). Both energy deficit and oxidative stress lead to brain (and extra brain) cell destruction.

Given that the levels of lactate in the plasma and CSF compartments correlate in AD and FTD while those of pyruvate in plasma and CSF only correlate in AD, we postulate that plasma supplies these substrates to AD and FTD brains. The supply of lactate is not only extremely useful during exercise (Ide et al., 1999, 2000), in which the brain increases lactate uptake, but also maintains cerebral blood flow regulation as far as possible (Hein et al., 2006; Yamanishi et al., 2006; Gordon et al., 2008).

### 4.3. Potential impact of the study metabolic alterations on neuroplasticity, synapse plasticity, and transmission

**FIGURE 1:**
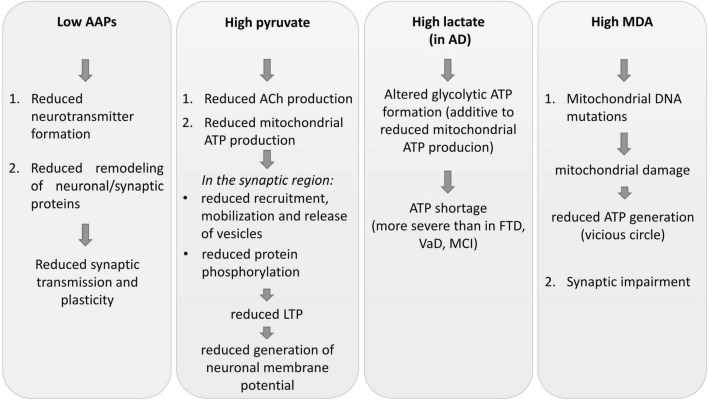
Some impacts of CSF metabolic alterations on neuronal plasticity, synaptic plasticity, and transmission. AAPs, amino acid precursors; AD, Alzheimer’s disease; FTD, frontotemporal dementia; MCI, mild cognitive impairment; VaD, vascular dementia; MDA, malondialdehyde; ACh, acetylcholine; LTP, long-term potentiation; ATP, adenosine triphosphate.

The results of this study suggest that metabolic alterations may potentially lead to the worsening of brain structural and functional changes and to the progression of cerebral damage (Figure 1).

Low AAPs indicate that the lack of neurotransmitter formation, the first step of neurochemical transmission processes, may contribute to deficits in synaptic transmission. The reductions of the essential Trp, Phenyl-, and Met can impair the structure and function of the neuronal network (Shepherd and Huganir, 2007; Bramham, 2008) by limiting the rate of protein synthesis and activation, in particular for the remodeling of synapse plasticity.

Altered synapse proteins could be responsible for alterations of the synaptic response to subsequent neurotransmitter release (Todorova and Blokland, 2017), reduced dendritic and spine growth, and synaptogenesis (Wong and Ghosh, 2002; Mattson et al., 2008).

Brain mitochondria dysfunction and reduced energy production (ATP), as inferred by pyruvate accumulation, reduces synaptic function and plasticity that normally depend on mitochondria (Cheng et al., 2010; Amaral and Pozzo-Miller, 2012; Ivannikov et al., 2013; Manczak et al., 2013; Brot et al., 2014; Su et al., 2014).

Moreover, deficit in ATP generation does not support (or only to a very small degree) synaptic vesicle recruitment and release, protein phosphorylation processes (Stefani et al., 1997; Attwell and Laughlin, 2001; Verstreken et al., 2005; Valenti et al., 2014), neurotransmitter release via vesicle exocytosis and mobilization, or dendritic remodeling (Cheng et al., 2010; Valenti et al., 2014).

Inadequate mitochondrial ATP production impairs long-term-potentiation (LTP) (Cunha et al., 1996), a well-documented experimental model of learning and memory (Morris, 2013).

Brain ATP deficit reduces the development of neuronal pre- and post-synaptic compartments, the generation of membrane potential, synaptic vesicle recruitment and release, and protein phosphorylation activities (Stefani et al., 1997; Attwell and Laughlin, 2001; Verstreken et al., 2005; Valenti et al., 2014).

**FIGURE 2:**
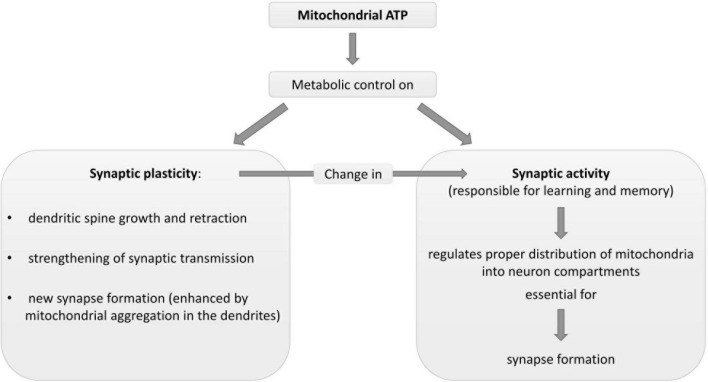
Description of some physiological metabolic interrelationships between neuronal mitochondria and synapse plasticity and activity. ATP, adenosine triphosphate.

As mitochondrial and synaptic plasticity influence each other (Figure 2; Amaral and Pozzo-Miller, 2012), deficits in synaptic plasticity lead to reduced mitochondrial biogenesis. Indeed, some signaling pathways regulating neuroplasticity, such as estrogen, growth factors, and nitric oxide (Foy et al., 2008; Brinton, 2009; Harooni et al., 2009; Minichiello, 2009), change mitochondrial formation (Williams et al., 1998; Gutsaeva et al., 2008; Klinge, 2008; Renton et al., 2010). Impaired synaptic activation and LTP formation (Mattson and Liu, 2003) can reduce mitochondrial energy production, Ca^2+^ pump activity and gene expression.

Mitochondria damage-increased ROS and high oxidative stress expose both demented patients and MCI subjects to DNA mutations (Mattson and Liu, 2003) and mitochondrial damage (vicious circle). Oxidative stress is associated with synaptic impairments, in particular in aging (Brawek et al., 2010). This contributes to explaining the cognitive impairment in the MCI subjects in the current study.

### 4.4. Limitations

There is a need for future investigations to overcome the limitations of this study.

Other common neurodegenerative dementias such as Parkinson’s disease dementia and dementia with Lewy bodies were not considered in this study.

The results of the study should be confirmed in a larger population of FTD, VaD patients, and MCI subjects. We believe this is especially important for CSF lactate levels. Indeed, in the current investigation, we also found high CSF lactate levels in FTD, VaD patients and MCI subjects although they were not statistically significant, probably because of the small sample sizes.

Another limit of the study is that the brain magnetic resonance imaging was performed, but due to differences in protocols could not be used to draw any correlation and analysis.

In the current study we did not analyze and discuss other signaling molecules including glutamate, which plays a critical role in synaptic plasticity and energy cellular metabolism (Mattson et al., 1995; Trenkner et al., 1996) by increasing mitochondrial oxygen consumption and thereby ATP production (Schuchmann et al., 2005; Mattson, 2007). In our opinion, possible differences in CSF glutamate among the dementia subtypes and between the dementia subtypes and MCI may be particularly important.

### 4.5. Future research

The results of the current and previous studies (Aquilani et al., 2020, 2022) may raise the question of whether oral supplementation with a specific cluster of AAs including adequate amounts of Tyr, Trp, Phenyl-, and Met could improve patients’ levels in the CSF compartment while reducing CSF pyruvate accumulation in patients with AD, FTD, and VaD, and subjects with MCI. Moreover, it may be important to document whether the addition of antioxidant agents to the AA cluster could reduce brain oxidative stress.

## 5. Conclusion

The study shows that similar brain reductions in the amino acid precursors of cholinergic, adrenergic, serotonergic neurotransmitters, altered energy substrates, and increased oxidative stress may be shared by patients with Alzheimer’s disease, vascular, and frontotemporal dementias, and subjects with mild cognitive impairment.

## Data availability statement

The raw data supporting the conclusions of this article will be made available by the authors, without undue reservation.

## Ethics statement

The study was approved by the Local Ethical Committee of Ospedale San Raffaele-Istituto di Ricovero e Cura a Carattere Scientifico-Milano, Italy (Project identification code: MAIR2016; Ethical Approval: 130/INT/2016; date: 8 September 2016). The studies were conducted in accordance with the local legislation and institutional requirements. The participants or their legal representatives provided their written informed consent to participate in this study.

## Author contributions

RA, AC, and MV contributed to the conception and design of the study. PI, GP, MB, MV, and MD contributed to the methodology. FB, DB, ED, MC, and AB contributed to the acquisition of data for the study. RM performed the statistical analysis. RA contributed to the interpretation of data for the study and wrote the first draft of the manuscript. MV and MC wrote sections of the manuscript. All authors contributed to the manuscript revision, and read and approved the submitted version.
